# Supplemental Sugar Is Required for Sex Pheromone Biosynthesis in *Mythimna separata*

**DOI:** 10.3389/fphys.2020.605145

**Published:** 2020-12-18

**Authors:** Yaling Zhang, Yuanchen Zhang, Shuangyan Yao, Gaoping Wang, Jizhen Wei, Mengfang Du, Shiheng An, Xinming Yin

**Affiliations:** ^1^Collaborative Innovation Center of Henan Grain Crops, College of Plant Protection, Henan Agricultural University, Zhengzhou, China; ^2^College of Biology and Food Engineering, Innovation and Practice Base for Postdoctors, Anyang Institute of Technology, Anyang, China

**Keywords:** *Mythimna separata*, sugar feeding, trehalase, sex pheromone biosynthesis, starve

## Abstract

Supplemental nutrients of adult moths maximize moth fitness and contribute to the pollination of many plants. Previous reports have revealed that sugar feeding promotes to sex pheromone biosynthesis by increasing the haemolymph trehalose concentration in mating moths. Here, *Mythimna separata* adults were employed as a model to investigate the effect of sugar feeding on sex pheromone biosynthesis. Results showed that in virgin females, sugar feeding markedly increased the concentrations of trehalose, pyruvic acid, and acyl-CoA in pheromone glands (PGs), which in turn led to an increase in sex pheromone titer, female ability to attract males and successfully mating frequency in sugar-fed females. Consistently, sugar-fed females laid more eggs than water-fed females. Furthermore, the refeeding of starved females also caused significantly increase in the concentrations of trehalose, pyruvic acid, and acyl-CoA in PGs, thus facilitating a significant increase in sex pheromone production. Most importantly, RNAi-mediated knockdown of trehalase (leading to PG starvation) resulted in an increase in trehalose content, and decrease in the concentrations of pyruvic acid, and acyl-CoA in PGs, which in turn led to a decrease of sex pheromone titer, female ability to attract males and successful mating efficacy. Altogether, results revealed a mechanism by which sugar feeding contributed to trehalose utilization in PGs, promoted to significantly increased sex pheromone precursor by increasing the concentrations of pyruvic acid and acyl-CoA, and facilitated to sex pheromone biosynthesis and successful mating.

## Introduction

Insects are the most prosperous species on earth. Strong reproductive capacity is one of important factors for insect prosperity. Once the environment is suitable, insects produce a huge quantity of offspring, which causes the population to increase rapidly in a short period, finally leading to pest outbreak and considerable loss of crops. Like that of animals, the reproduction of most insects is sexual mating. Therefore, successful mating plays an important role in the process of insect reproduction. Unlike higher animals, insects, especially Lepidopteran moths, usually employ female sex pheromones as chemical signals for long-distance information exchange (Ando et al., 2004), which is the key factor for male moths to locate female moths.

Sex pheromone is a micro chemical synthesized and released by the female sex pheromone glands (PGs) located between the 8th and 9th abdominal segments (Tillman et al., 1999; Jurenka, 2003). Sex pheromone is comprised of multicomponent unsaturated fatty acids (10–18 straight chain hydrocarbons) with oxidized ends (alcohols, aldehydes, acetates, etc.) (Tillman et al., 1999). Sex pheromones were *de novo* synthesized from acetyl-CoA followed by chain shortening, desaturation and reduction (Tillman et al., 1999; Jurenka, 2003). Studies had confirmed that a neuropeptide, named as pheromone biosynthesis activating neuropeptide (PBAN), regulated female pheromone biosynthesis and release in Lepidoptera (Tillman et al., 1999). PBAN gene was first identified in *Bombyx mori* and *Helicoverpa zea* (Raina et al., 1989; Kitamura et al., 1990), and then in other Lepidoptera species (Davis et al., 1992; Ma et al., 1994; Xu et al., 1995; Kochansky et al., 1997; Jacquin-Joly et al., 1998; Tillman et al., 1999; Iglesias et al., 2002; Rafaeli, 2002; Ando et al., 2004; Xu and Denlinger, 2004; Lee and Boo, 2005; Jing et al., 2007). Sequence analysis found that PBAN consists of 33 amino acid residues with a conserved amidated C-terminal (Kochansky et al., 1997). PBAN receptor was also identified in *H. Zea* and *B. mori* (Choi et al., 2003; Hull et al., 2004). Studies confirmed that PBAN receptor was a kind of conserved G protein receptor (Choi et al., 2003). The finding of PBAN receptor was a groundbreaking contribution to the deciphering of signal transduction cascade mechanism following the ligand PBAN binding to its respective PBAN receptor in the PG. PBAN signal cascades were well documented in subsequent studies. In *H. armigera*, after binding with its receptor located at the plasma membrane of PG cells, PBAN employed Ca^2+^ and cAMP as second messages. On the one hand, Ca^2+^ -calmodulin complexes activate calcineurin, which in turn activate acetyl-CoA carboxylase through dephosphorylation at ser92 site (Acetyl-CoA carboxylase is the critical enzyme in fatty acid biosynthesis, which catalyzes carboxylation of acetyl-CoA to yield malonyl-CoA), finally leading to sex pheromone biosynthesis and release (Du et al., 2017). On the other hand, the cAMP/PKA signal suppresses the AMPK activity, which is an upstream kinase of acetyl-CoA carboxylase, thus ensuring the calcineurin activation of acetyl-CoA carboxylase (Du et al., 2017). In *B. mori*, PBAN was found to use Ca^2+^ as a second message. Ca^2+^ first activated calcineurin (a protein phosphatase) and calmodulin-dependent kinase II (CamKII). And then calcineurin in turn mediated fatty acyl reductase, while CamKII activated lipid storage droplet protein-1 by phosphorylation, thereby leading to lipolytic release of stored pheromone precursors (Ohnishi et al., 2011). These results well deciphered the detailed mechanism of PBAN signal transduction.

PBAN functions as an endogenous regulator of sex pheromone biosynthesis and release in moths. In fact, the biosynthesis and release of sex pheromone are also affected by other endogenous factors. Studies found that in *H. zea*, a pheromonostaitc peptide secreted from male accessory glands could strongly inhibit sex pheromone biosynthesis (Kingan et al., 1995). In *Drosophila melanogaster*, a sex peptide (SP) generated from the male accessory glands caused strong suppression of female receptivity, leading to a few mating of females with males (Ottiger et al., 2000). Most interestingly, this SP from *D. melanogaster* also promoted juvenile hormone synthesis and eventually caused a decrease in sex pheromone production in *H. armigera* (Fan et al., 2000). In addition, some exogenous factors also affect sex pheromone biosynthesis. In *H. virescens*, sugar-feeding led to a significant increase in sex pheromone biosynthesis in mating females, further studies demonstrated that sugar-feeding contributed to the increase in hemolymph trehalose, a key material to generate acetyl-CoA *via* glycolysis and tricarboxylic acid cycle (Foster, 2009). Most importantly, 65% of sex pheromone production comes from a single adult feed, implying the importance of sugar feeding on sex pheromone biosynthesis (Foster and Anderson, 2015). These studies had considerably deepened the understanding on the supplemental nutrition of female adults. In the present study, *M. separata* was used as a model to investigate the effect of adult supplemental nutrition on female mating, especially on sex pheromone biosynthesis.

## Materials and Methods

### Insects

*M. separata* larvae were collected from maize field in Jiyuan, Henan province, China. Larvae were maintained with an artificial diet at 28°C with 16 h light/8 h dark photoperiod. Pupae were distinguished by gender and stored in separate cages until emergence. Newly emerged adults in the 1st photophase were referred to as 1 day-old adults and were subjected to subsequent feeding experiment.

### Chemicals

PBAN was biosynthesized from Sangon Biotech (Shanghai, China). Z11-hexadecenal (Z11–16Ald), sex pheromone component of *M. separata*, was obtained from Sigma (St. Louis, MO) and used to quantify sex pheromone in PG by gas chromatography/mass spectrometry (GC/MS).

### The Determination of Trehalose, Pyruvic Acid, and Acyl-CoA Concentration in the PGs

Newly emerged females were collected and placed on a new cage with 5% sugar solution (*n* ≥ 24). The corresponding control females were also placed on other new cage with water alone. PGs were collected at different time points after feeding with sugar (including 48, 72, and 96 h after feeding with sugar). For starving treatments, the *n* ≥ 20 newly emerged females (for each treatment) were fed only water for 24, 48, and 72 h later, then refed with 5% sugar solution. PGs were collected 24 h (including one photophase and one night phase) after refeeding to keep the adults had enough time to feed sugar, and biosynthesize sex pheromone. The above harvested PGs were subjected to quantitative determination of the concentrations of trehalose, pyruvic acid, and acyl-CoA by using the concentration determination kits (A149-1-1, A076-1-1, and A081 kit from Nanjing Jiancheng Bioengineering Institute, respectively). The detailed steps followed the manufacturer’s suggestions^1^ and the previous reports (Du et al., 2017; Jiang et al., 2020; Xu et al., 2020).

### The Measurement of Sex Pheromone Production by GC/MS

Newly emerged females were collected and placed on 5% sugar solution (*n* ≥ 20). The corresponding control females were on water alone. Three replicates were adopted. Prior to collecting PGs, 10 pmol of PBAN was injected into PGs, after 1 h waiting time, PGs were collected at different time points after feeding (including 48, 72, and 96 h after feeding). The harvested PGs were dissolved in hexane and then subjected to further GC/MS analysis as described above (Zhao et al., 2018).

For starving treatment, the *n* ≥ 20 newly emerged females (for each treatment) were fed only water for 24, 48, and 72 h later, then refed with 5% sugar solution for 24 h, PGs were then collected as the above method, and sex pheromone productions were tested by GC/MS.

### Female Ability to Attract Males

Olfactometer was employed to investigate the effect of sugar feeding on female ability to attract males (Zhang et al., 2018). 20 newly emerged females were fed with 5% sugar solution or water. 72 h later, two treated females (5% sugar or water) were placed in two capture chambers (28 cm height × 30 cm width × 30 cm length with a 6 cm diameter of hole), respectively. 100 males (2 day-old) were together placed in released chamber (32 cm height × 30 cm width × 60 cm length) on 10 p.m. After 12 h (20:00 p.m. to 8:00 a.m.) waiting time, the male numbers in two capture chambers were recorded.

### Mating Behavior

Females (*n* = 30) with sugar feeding at different time points (48, 72, and 96 h after feeding) were place a new cage (40 cm × 40 cm × 40 cm) with same number of males for 24 h. Then, the females were dissected, the proportion of successful mating was determined according to the presence of spermatophore in the bursa copulatrix (Zhao et al., 2018). Three biological replicates of each treatment were done.

### Fecundity

Newly emerged females (*n* ≥ 20) were collected and placed on a new cage with same number of males. In addition, 5% sugar solution was placed on cage to meet the female need to sugar. The corresponding control females were on water alone. Egg numbers were recorded after 120 h feeding. Three biological replicates of each treatment were done.

### Quantitative Real-time PCR

Total RNA was extracted from the collected PG samples using TRIzol reagent (Invitrogen, Carlsbad, CA, United States) according to the manufacturer’s instructions. RT-PCR was performed on by using first-strand cDNA as template, which was obtained from 1 μg total RNA exacted from each PG sample using the PrimeScriptRT reagent kit with gDNA Eraser (Takara, Beijing, China). The relative expression of gene associated sex pheromone biosynthesis (Du et al., 2017), trehalose transporter (Trel-E) (GenBank: MT995929), fatty acid reductase (FAR) (GenBank: MT995931), acetyl CoA carboxylase (ACC) (GenBank: MW286766), △-11 desturase (GenBank: MW286764), hexokinase (HK) (GenBank: MT995930), pyruvate kinase (PK) (GenBank: MW286765), and trehalase (GenBank: MT995933) were tested. The primers used for RT-PCR analysis are listed in Table 1. *M. separata* ribosomal protein gene GAPDH (GenBank: MT995932) and β-actin (GenBank: GQ856238.1) genes were used as reference genes. Real-time SYBR Green Supermix (Takara, Beijing, China) was employed to run PCR on an Applied Biosystems 7,500 Fast Real-Time PCR system (ABI, Carlsbad, CA, United States) following the manufacturer’s instructions. The thermal cycler conditions for RT-PCR were set as following: 95°C for 4 min, followed by 40 cycles of 95°C for 15 s and 60°C for 20 s. Melting curve analysis and agarose gel electrophoresis analysis were employed to analysis the specificity of the SYBR Green PCR signal. The reliability and reproducibility of RT-PCR results were ensured by employing three biological replicates of each sample. Melting curve analysis and the calculation method for expression level of the target genes was done according to the description in Liu et al. (2015) and Wei et al. (2019).

**TABLE 1:** Primers used for Real-time PCR in this study.

**Primer name**	**Strand orientation**	**Sequence (5′–3′)**
GAPDH –RTF	Forward	AGAGGGTGGTGCCAAGAAG
GAPDH –RTR	Reverse	GTAGCGGTGGTAGCGTGTA
β-actin –RTF	Forward	ACGAACGATTCCGTTGCCCT
β-actin –RTR	Reverse	TCTGCATACGGTCGGCGATG
Trel-E-RTF	Forward	AGTTATGTATGCTGCCTTTG
Trel-E –RTR	Reverse	TATGCTGTTGAGTTCGGTAA
FAR-RTF	Forward	AGTATCCATCGTCTTCCAT
FAR-RTR	Reverse	TCAACACTTCGTAGTCAGG
ACC –RTF	Forward	CACCTTTATGCTGCTTATC
ACC –RTR	Reverse	GTCTGTTACTTCTTGTCCCT
△-11 desturase -RTF	Forward	ACGGGCTTTATCTGTGCTT
△-11 desturase -RTR	Reverse	CAGTCAATGGCGGAGTTTT
Hexokinase -RTF	Forward	ATCTCACGCAACTGAAGCA
Hexokinase -RTR	Reverse	GCCGTCCTCTGACAGCATC
PK-RTF	Forward	GTAAAGAAGCCTCGTCCCA
PK-RTR	Reverse	CGTTGAAGAGCTGCCTGTG
Trehalase-RTF	Forward	TACAGTTTAGTTCCGCTT
Trehalase-RTR	Reverse	CCGTTAGGGATGTGACCG

### Double-Stranded RNA (dsRNA) Synthesis

The templates for dsRNA synthesis were amplified using gene-specific primers containing T7 polymerase sites following previously described methods (Du et al., 2012a,b). A 488 bp cDNA fragment of trehalase was PCR-amplified to generate the template for *in vitro* dsRNA synthesis. The primers were forward primer (5′-GATCACTAATAC GACTCACTATAGGGAGAAGCCACCTATGTTGACAGC-3′) and reverse primer (5′-GATCACTAATACGACTCACTATAGGG AGACAAATCTGAGGCTAACGCTG-3′). PCR product was purified and then used as the template for *in vitro* dsRNA synthesis. MEGAscript RNAi kit (Ambion, Vilnius, Lithuaria) was used to synthesized dsRNA following the manufacturer’s instructions. Resulting synthesized dsRNA was first treated with DNase and RNase to remove template DNA and single-stranded RNA and then purified using MEGAclearTM columns (Ambion, Vilnius, Lithuaria). Resulting dsRNA was finally eluted in diethyl pyrocarbonate (DEPC)-treated nuclease-free water. The concentrations of dsRNA were measured using a biophotometer (Eppendorf). Correspondingly enhanced green fluorescent protein (EGFP) dsRNA was also generated according to the above mentioned method and was used as the control (Du et al., 2012a,b).

### The Effect of Trehalase Knockdown on Sex Pheromone Biosynthesis and Mating

dsRNA (10 μg) was injected into the intersegment membrane between the 7th and 8th abdominal segments of newly emerged females. 48 h after injection, the PGs were harvested and subject to RT-PCR analysis to test the interference frequency of RNAi. Just as above mentioned, females injected with dsRNA were decapitated to remove endogenous PBAN. 48 h after dsRNA injection, 10 pmol of PBAN was injected into female body. After 1 h waiting time, PGs were collected and dissolved in 100 μL hexane and stored in −80°C followed by GC/MS analysis to measure sex pheromone production. Females injected with 10 μg dsEGFP RNA were used as the controls. Correspondingly, the concernations of trehalose, pyruvate and acetyl-CoA were measured according to above mentioned methods to analysis the effect of trehalose knockdown on the concernations of trehalose, pyruvate and acetyl-CoA.

As the above method, 20 newly emerged females were injected with 15 μg dsRNA (trehalase or EGFP). 72 h after dsRNA injection, Male choosing number were recorded 12 h later. 30 females injected with dsRNA (trehalase or EGFP) were place a new cage with same number of untreated males. After 72 h, the proportion of successful mating was determined according to the presence of spermatophore in the bursa copulatrix (Zhao et al., 2018). Three biological replicates of each treatment were done.

### Statistical Analysis

Significant difference in each two different treatments was compared with Student’s *t*-test. Statistically significant differences are denoted with ^∗^ (*P* < 0.05), ^∗∗^ (*P* < 0.01), and ^∗∗∗^ (*P* < 0.001). A *Yate-corrected chi-square test* was employed to determine the difference between the frequencies of male choosing different females with *P* < 0.001 (Zhang et al., 2018).

## Results

### Effect of Sugar Feeding on the Contents of Trehalose, Pyruvic Acid, and Acyl-CoA in PGs

**FIGURE 1:**
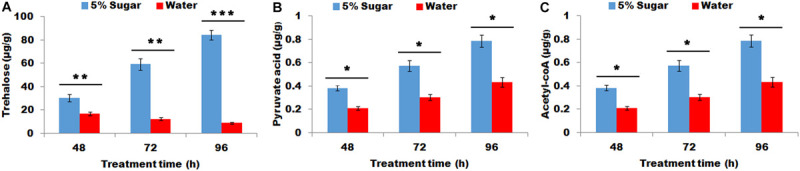
Effect of sugar feeding on contents of trehalose **(A)**, pyruvic acid **(B)**, and acyl-CoA **(C)** in PGs. Each error bar represents the standard error of the mean from three replicates. Significant difference between 5% sugar feeding and water feeding was compared with Student’s *t*-test. Asterisks showed significant differences, * (*P* < 0.05), ** (*P* < 0.01), and *** (*P* < 0.001) (Student’s *t*-test).

The effect of sugar feeding on the contents of trehalose, pyruvic acid, and acyl-CoA were investigated. The results showed that sugar feeding contributed to a significant increase in trehalose, (*P* < 0.01, Figure 1A), pyruvic acid (*P* < 0.05, Figure 1B), and acyl-CoA (*P* < 0.05, Figure 1C) production in female PGs compared with water feeding, indicating that sugar feeding affects the production of trehalose, pyruvic acid, and acyl-CoA in PG tissues.

### Effect of Sugar Feeding on Sex Pheromone Production, Female’s Ability to Attract Males and Mating

**FIGURE 2:**
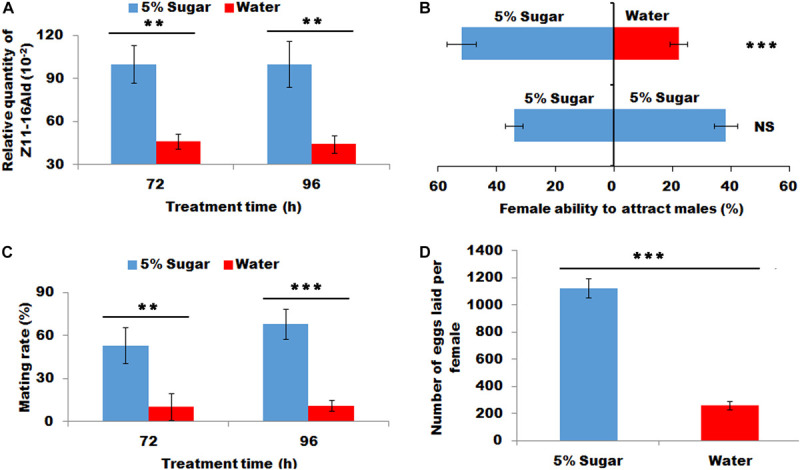
Effect of sugar feeding on sex pheromone production **(A)**, female ability to attract males **(B)**, mating rate **(C)**, and fecundity **(D)**. Each error bar represents the standard error of the mean from three replicates. Significant differences between 5% sugar feeding and water feeding in sex pheromone production, mating rate and fecundity were compared with Student’s *t*-test. The significant difference between 5% sugar feeding and water feeding in attractant rate was compared with *Yate-corrected chi-square test*. Asterisks showed significant differences, ** (*P* < 0.01) and *** (*P* < 0.001) (Student’s *t*-test).

The sugar-fed females released significantly more sex pheromone at 72 and 96 h than water-feeding females (*P* < 0.01, Figure 2A). This increased sex pheromone production significantly lured more males than those fed with water (*X*^2^ = 11.36, *P* < 0.001, Figure 2B). Most importantly sugar feeding promoted to an increase in the proportion of females to mate at 72 and 96 h (*P* < 0.01 and *P* < 0.001, respectively, Figure 2C), indicating successful mating frequency in sugar-fed females. Consistently, the sugar-fed females laid more eggs than the water-fed females (*P* < 0.001, Figure 2D).

### Effect of Sugar Re-feeding of Starved Female on Sex Pheromone Biosynthesis

**FIGURE 3:**
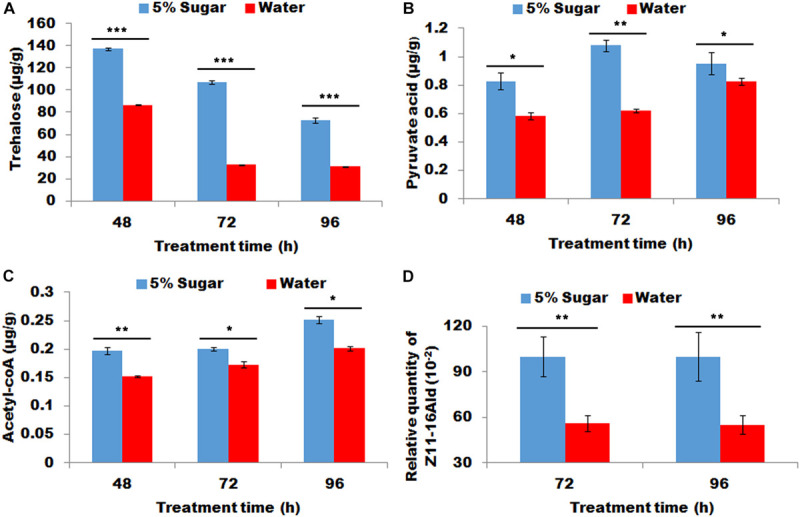
Effect of sugar re-feeding of starved female on contents of trehalose **(A)**, pyruvic acid **(B)**, and acyl-CoA **(C)** and sex pheromone production **(D)**. As for 48 h treatment, newly-emerged females were first fed with water for 24 h and then refed with 5% sugar solution for 24 h (corresponding control females continue to feed with water for 24 h). As for 72 h treatment, newly-emerged females were first fed with water for 48 h and then re-fed with 5% sugar solution for 24 h (corresponding control females continue to feed with water for 24 h). As for 96 h treatment, newly-emerged females were first fed with water for 72 h and then re-fed with 5% sugar solution for 24 h (corresponding control females continue to feed with water for 24 h). Each error bar represents the standard error of the mean from three replicates. Significant difference between 5% sugar feeding and water feeding was compared with Student’s *t*-test. Asterisks showed significant differences, * (*P* < 0.05), ** (*P* < 0.01), and *** (*P* < 0.001) (Student’s *t*-test).

Starved females re-fed with sugar demonstrated significantly more contents of trehalose, pyruvic acid, and acyl-CoA in PG tissues than starved females fed with water (*P* < 0.01, Figures 3A–C). Most importantly, sugar refeeding of starved females caused to a significant increase in sex pheromone production (*P* < 0.01, Figure 3D).

### Effect of Sugar Feeding on the Relative Expression of Genes Associated With Sex Pheromone Biosynthesis

**FIGURE 4:**
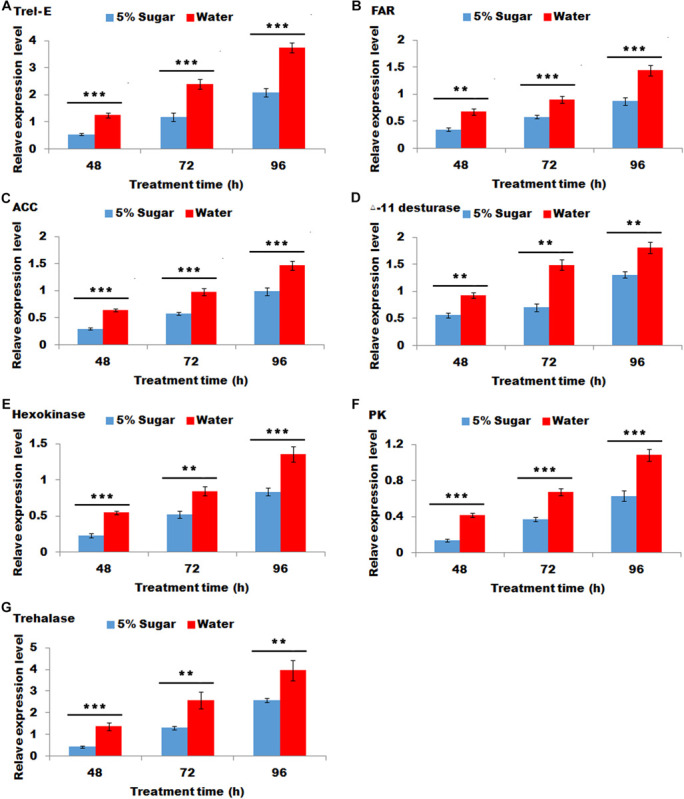
Effect of sugar feeding on the relative expression levels of genes associated with sex pheromone biosynthesis. Each error bar represents the standard error of the mean from three replicates. Significant differences between 5% sugar feeding and water feeding in the expression of Trel-E **(A)**, fatty acid reductase (FAR) **(B)**, ACC **(C)**, △-11 desturase **(D)**, hexokinase (HK) **(E)**, pyruvate kinase (PK) **(F)**, and trehalase **(G)** were compared with Student’s *t*-test. Asterisks showed significant differences, ** (*P* < 0.01) and *** (*P* < 0.001) (Student’s *t*-test).

Sugar feeding led to a significant decrease in the transcripts of Trel-E, fatty acid reductase (FAR), ACC, deta-11 desturase, hexokinase (HK), pyruvate kinase (PK) and trehalase after 48, 72, and 96 h (Figure 4), indicating that these gene expression levels were negative response to sugar feeding.

### Effect of Trehalase Knockdown on Sex Pheromone Biosynthesis, Female Ability to Attract Males and Mating

**FIGURE 5:**
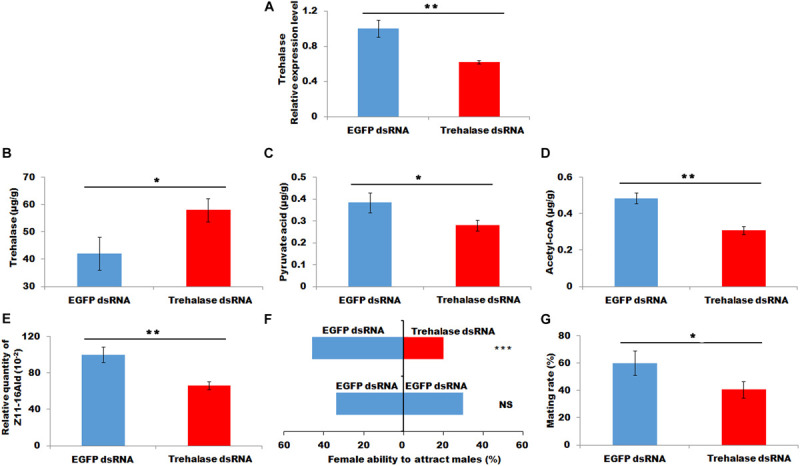
Effect of trehalase knockdown on contents of trehalose **(B)**, pyruvic acid **(C)**, and acyl-CoA **(D)**, sex pheromone production **(E)**, attractant rate **(F)**, and mating rate **(G)**. **(A)**, the relative expression of trehalase. Each error bar represents the standard error of the mean from three replicates. Significant differences between injecting with trehalase RNA and dsEGFP RNA in expression level of trehalase, content of trehalose, pyruvic acid and acyl-CoA, sex pheromone production, attractant rate and mating rate were compared with Student’s *t*-test. The significant difference in attractant rate was compared with *Yate-corrected chi-square test*. Asterisks showed significant differences, * (*P* < 0.05), ** (*P* < 0.01), and *** (*P* < 0.001) (Student’s *t*-test).

A double-strand RNA of trehalase was injected into newly emerged females. RT-PCR was employed to investigate the interference efficacy at 48 h after injection. The results demonstrated that the injection of trehalase dsRNA led to a significant decrease in the transcript level of trehalase (*P* < 0.01, Figure 5A). After successful knockdown, the decrease in trehalase transcript caused a significant increase in trehalose in PGs (*P* < 0.05, Figure 5B). Consistently the RNAi-mediated knockdown of trehalase also resulted in a significant reduction in the contents of pyruvic acid and acytl-CoA (*P* < 0.05 and *P* < 0.01, respectively, Figures 5C,D). In addition, the females injected with trehalase dsRNA showed significantly decreased sex pheromone production, female ability to attract males and mating efficacy (*P* < 0.05, *X*^2^ = 9.47, *P* < 0.001 and *P* < 0.05, respectively, Figures 5E–G), compared with the control females injected with EGFP dsRNA.

## Discussion

Insect larvae must feed its hosts that provided necessary nutrients to meet the needs of their growth and development (O’Brien et al., 2002). Therefore, feeding in larval stage was considered to be crucial for insect growth and development. As for adults, the food resources for the reproductions of most insects were thought to depend on larval feeding (Bradbury and Vehrencamp, 1998). However, most Lepidopteran species feed nectar that consists of water and sugar in the adult stage. Sugar feeding in the adult stage maximized the capacity of adult reproduction; however, it was generally thought to have a little effect on sex pheromone production (Bradbury and Vehrencamp, 1998). For example, in *Heliconius melpomene*, the male pheromone composition depends on larval feeding and is not associated with adult diet (Darragh et al., 2019), similarly a number of arctiid moth species produced their pheromones by using materials accumulated from larval feeding (Schulz et al., 1993; Edgar et al., 2007). These studies showed that adult moths seem to not use adult feeding to produce sex pheromone. However, a previous study revealed that the sugar feeding significantly increase sex pheromone production in mated *H. virescens* (Foster, 2009). A subsequent study also revealed that even in virgin moths of *H. virescens*, adult feeding significantly increased sex pheromones, indicating that the starved stress in adults (sugar deficiency) led to a significant decrease in sex production on virgin or mated moths (Foster and Johnson, 2010). Most importantly, in *H. virescens* moths, only a single feeding (glucose) contributed to a maximum of 65% of *de novo* biosynthesized pheromone production (Foster and Anderson, 2015), indicating that sugar supplement in adult moths played a crucial role in sex pheromone biosynthesis. These results clarified the importance of supplemental sugar in sex pheromone biosynthesis. In the present study, sugar feeding significantly increased sex pheromone production and promoted to more proportion females to mate in *M. separata* (Figures 1–3). This finding was consistent with the result from *H. virescens* moths, that was, supplementary sugar played an important role in sex pheromone biosynthesis.

Sex pheromones were biosynthesized from precursor acetyl-CoA *via* fatty acid synthesis followed by desaturation, chain-shortening and reductive modification of carbonyl carbon (Tillman et al., 1999). As the precursor of sex pheromones, acetyl-CoA was produced *via* glycolysis and tricarboxylic acid cycle (Foster and Anderson, 2015). Most acetyl-CoAs for fatty acid biosynthesis originated from citrate, generated from pyruvate or fatty acid oxidation in the mitochondria. As for adult moth, carbohydrate was a supplementary sugar, which could be transformed to trehalose rapidly and easily. Trehalose serves as the main material to produce acetyl-CoA *via* glycolysis and tricarboxylic acid cycle (Foster and Anderson, 2015). In *H. virescens* moths, the deprivation of adult feeding caused a significant decrease in the trehalose concentration in adult haemolymph, finally leading to a reduction in sex pheromone production (Foster and Anderson, 2015). In *M. separata* adult, trehalose concentration in adult haemolymph was not detected in present study. Correspondingly, the trehalose concentration in PG tissues was investigated because trehalose in PG was better indicator for sex pheromone precursor than the trehalose in haemolymph. The results showed that sugar feeding increased trehalose concentration in PG tissues (Figures 1A, 3A). Most importantly, the increase in the trehalose concentration in PG tissues in turn promoted an increase in pyruvate and subsequent acetyl-CoA production *via* glycolysis and tricarboxylic acid cycle, respectively (Figures 1B,C, 3B,C), finally facilitating sex pheromone biosynthesis (Figures 2B, 3D). Thus the trehalose in adult haemolymph contributed to sex pheromone biosynthesis and release in *M. separata* as shown by the trehalose, pyruvate and acetyl-CoA production in PG tissues. Given that mating leads to a great demand for sugar, sugar feeding in mated female contributes to a significant increase in trehalose, pyruvate, acetyl-CoA and subsequent sex pheromones in *M. separata* moths. A similar phenomenon was also found in *H. virescens* adult, in which sugar feeding in mated female promoted an increase in sex pheromone. Interestingly, the early feeding of adult *H. virecence* (3 days before) had no effect on sex pheromone production, only adult feeding after 6 days showed a significant effect on sex pheromone biosynthesis (Foster and Johnson, 2010). However, in *M. separata* moths, only 2 days (48 h) feeding had a significant effect on sex pheromone biosynthesis, indicating the importance of sugar supplement for sex pheromone biosynthesis in this species (Figure 1). Different from *H. virescense*, *M. separata* moths have the habits of migration and gluttony, frequently leading to a large-area outbreak. The feeding amounts of *M. separata* moths were dozens of times higher than those of other Lepidopteran moths, indicating that the demands for supplementary nutrition were very high in *M. separata* moths. This finding possibly explained the reason why only 2 days (48 h) feeding significantly increased the sex pheromone production.

*M. separata* is a kind of gluttonous insect, thus investigating the effect of sugar supplement on sex pheromone in adult *M. separata* is ideal. However, some adult moths have no habits of supplementary nutrition. For example, after emergence, *B. mori* adults do not feed on anything and immediately produce sex pheromones and then mate (Matsumoto et al., 2010). Thus, no supplementary nutrition is available, females have to utilize the nutrition obtained by larvae to produce sex pheromone. In this kind of pattern, precursor fatty acids were generated from acetyl-CoA by larval feeding, and then stored in PGs in the form of triacylglycerols (TAGs). Evidences also revealed that lipid drops began to accumulate 3 days before emergence and rapidly increased 1 day before the emergence in PG tissues (Matsumoto et al., 2010). Once emergence, TAGs were available following PBAN release (Ohnishi et al., 2011). Correspondingly, PBAN stimulation led to a rapid decrease in the amount of lipid drops (Ohnishi et al., 2011). Therefore, as for this kind of patter, PBAN regulated the lipolysis of TAGs stored in PGs in advance by larval feeding biosynthesis (Ohnishi et al., 2011). Different to this pattern, the precursor fatty acids in TAGs were unavailable in many adult moths, such as *Ostrinia nubilalis*, although the much higher accumulation of TAGs in PGs, the stored TAGs in PGs were a dead end store and they did not serve as precursor acids for sex pheromone biosynthesis (Ohnishi et al., 2011). In *H. virescens*, the TAGs in PGs are similarly unavailable for sex pheromone biosynthesis (Foster et al., 2017). In these moths, TAGs in PGs act as a buffer for fatty acyl CoA. Given that these TAGs are not used as sex pheromone biosynthesis, the females have to *de novo* biosynthesize the fatty acid precursor for sex pheromone biosynthesis (Foster et al., 2017). In this kind of pattern, a rapid and convenient method is to employ the hemolymph trehalose to obtain the required precursor fatty acid for sex pheromone, indicating that adult feeding probably plays an important role in the rapid biosynthesis of precursor fatty acid. Foster (2004, 2009), Foster and Johnson (2010), and Foster and Anderson (2015) provided irrefutable facts that supplementary sugar is the main material for the precursor fatty acids of sex pheromones. Even a single feeding (glucose) contributed to a maximum of 65% of *de novo* biosynthesized pheromone production (Foster and Anderson, 2015). Correspondingly, different from the regulated pattern in *B. mori*, PBAN regulated the steps of fatty acid synthesis in these species (Matsumoto et al., 2010). For example, in *H. armigera*, PBAN mediated the activation of acetyl-CoA carboxylase, a speed-limited enzyme of fatty acid biosynthesis, and facilitated sex pheromone biosynthesis (Du et al., 2017). Thus, the differences in the habits of adult feeding were consistent with the differences in PBAN actions.

In the present study, sugar feeding promoted the increase in trehalose, pyruvate, and acetyl-CoA production in *M. separata* (Figures 1, 3); and contributed to the increase in sex pheromone biosynthesis and the subsequent mating proportion of female moth (Figures 2, 3). Trehalase was chosen as a readout of sugar metabolism to further investigate the effect of sugar supplement on sex pheromone biosynthesis. Trehalase is a key enzyme that catalyzes the hydrolysis of trehalose into two glucose molecules (Becker et al., 1996). Trehalase knockdown means although trehalose is sufficient, it does not normally transform to glucose (Figure 5B). In other word, as adults continued to feed, the females were still in the state of starvation. The results of the present study were well agreement with the previous prediction. RNAi-mediated knockdown of trehalase led to a significant accumulation of trehalose in the PGs (Figure 5B), and a significant decrease in pyruvate, acetyl-CoA concentration and sex pheromone production in PGs (Figures 5C–E), finally led to a decrease in the female ability to attract males and female mating frequency (Figures 5F,G). The results showed that *M. separata* adult adopted same strategies with those of *H. virescens* to produce sex pheromone (Foster, 2009; Foster and Johnson, 2010). Thus, adult feeding (sugar) contributed to the biosynthesis of pyruvate and acetyl-CoA from trehalose, and increased the sex pheromone biosynthesis and subsequent mating frequency.

In our study, the mating of *M. separate* can be significantly affected by feeding sugar. As an important migratory agricultural pest, *M. separata* must obtain supplemental sugar to reach target habitats and lay eggs (Guo et al., 2018). Therefore, reasonable planting and layout of nectar sources along the migratory pathway may help to cut the sugar supply, and finally leading to failure in mating and reproduction of *M. separate*.

## Data Availability Statement

The raw data supporting the conclusions of this article will be made available by the authors, without undue reservation.

## Author Contributions

XY, JW, GW, MD, and SA conceived and designed the experiments and wrote the manuscript. YaZ, YuZ, SY, XY, JW, and SA conducted the experiments and analyzed the data. All authors read and approved the manuscript.

## Conflict of Interest

The authors declare that the research was conducted in the absence of any commercial or financial relationships that could be construed as a potential conflict of interest.

## Footnotes

1 http://www.njjcbio.com/
